# Electroconvulsive Therapy vs. Ketamine: A Scoping Review and Meta-Analysis of Rapid-Acting Interventions for Acute Suicidality in Adults

**DOI:** 10.1192/j.eurpsy.2026.11983

**Published:** 2026-07-31

**Authors:** V. Sharma, S. Ather, A. Sič, A. Asressahegn, A. Haryal, S. Prasad, T. Kainth, S. Gunturu

**Affiliations:** 1 Dayanand Medical College & Hospital, Ludhiana, India; 2 Psychiatry, SUNY Upstate Medical University, Syracuse NY, United States; 3 University of Belgrade, Belgrade, Serbia; 4 Saint Paul’s Hospital Millennium Medical College, Addis Ababa, Ethiopia; 5 Psychiatry, Texas Tech University Health Sciences Center, Odessa; 6 Psychiatry, BronxCare Health System, The Bronx, United States

## Abstract

**Introduction:**

Suicide is a leading cause of death in the psychiatric population. The existing modes of treatment (pharmacological and psychotherapeutic) for suicidality are effective but have longer onset, this delay causing a dangerous therapeutic gap in an acute crisis. We compare the two fast-acting treatments, the newer psychopharmacological agent Ketamine and well-established neuro-modulatory electroconvulsive therapy (ECT), for acute suicidality.

**Objectives:**

Comparing efficacy, safety and side effect profiles of Ketamine and electroconvulsive therapy through a scoping review and meta-analysis.

**Methods:**

PubMed was searched on 15 January 2025 without temporal and geographical restrictions, using keywords ‘Ketamine’, ‘Electroconvulsive therapy’ and ‘Suicidality’ yielding 916 records. After duplicate removal, 895 remaining records were screened and 52 relevant original research or meta-analysis were included as primary records. Non-original, non-English, non-human and not relevant were excluded (843 records). Additional 50 secondary sources were retrieved from 1) references of primary sources and 2) targeted open-source Google search while drafting the manuscript. The final 102 articles were subdivided into 3 subsections (30- ketamine + 57- ECT+ 15- comparison of modalities) for analysis. PRISMA-ScR guidelines were followed (Figure 1) and 5 different meta-analyses were conducted using RevMan 5.4.1.

**Results:**

Ketamine demonstrated high potential as an anti-suicidal agent. Clinical trials with suicidality as primary outcome showed significant improvement in scores on clinical suicidal scales with ketamine, with standard mean difference (SMD) of -1.24 (95% CI [-1.79, -.068], Z = 4.34, p < 0.0001), but the heterogeneity was high (I^2^= 92%). Trials with suicidality as secondary outcome also showed significant improvement with SMD -0.79 ([-1.00, -0.57], Z= 7.28, p< 0.00001; I^2^= 0%) (Figure 2). The research in last 10 years demonstrate that the risk of suicide was significantly decreased by 22% with ECT (overall hazard ratio: 0.78, 95% CI [0.68, 0.90], Z = 3.52, p = 0.0004; I^2=^ 0) (Figure 3). In psychopharmacological era, suicide and suicide attempt rates improved with ECT, with significant peto-odds ratio (OR) of 0.80 (95% CI [0.73, 0.88], Z = 4.85, p < 0.00001), but the heterogeneity was high (I^2^= 65%). Research from the shock therapy era was inconclusive with fallacious methodology. Ketamine has a rapid but short-lived effect with the side effects of dissociation, increased blood pressure, and nausea. In contrast, ECT requires multiple sessions, but a long-lasting impact, with cognitive side effects.

**Image 1:**
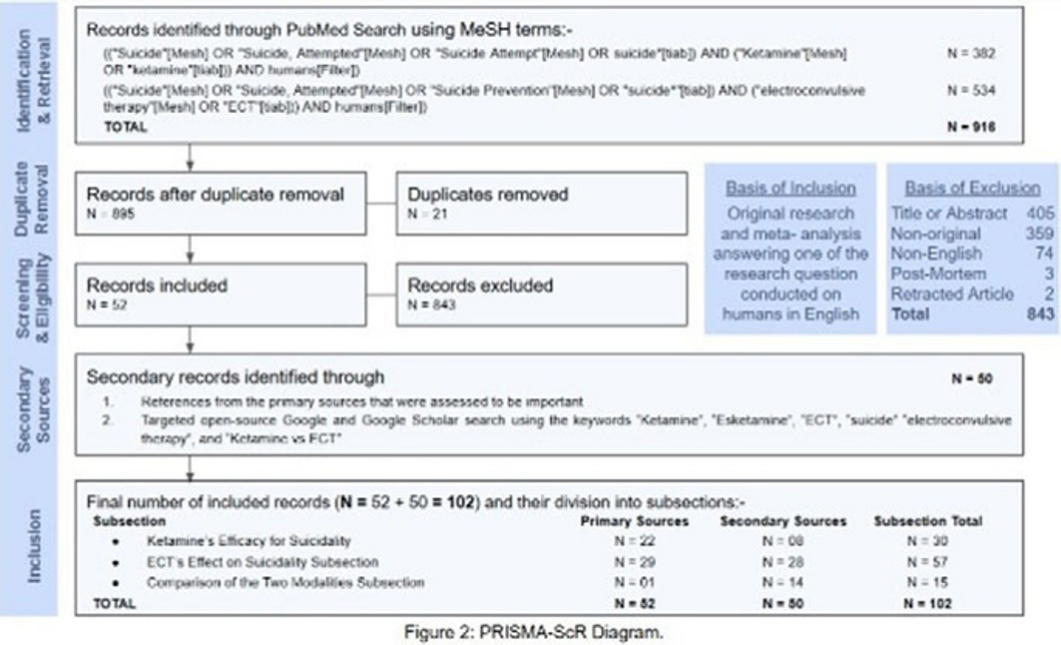
Image 1: Long description.

**Image 2:**
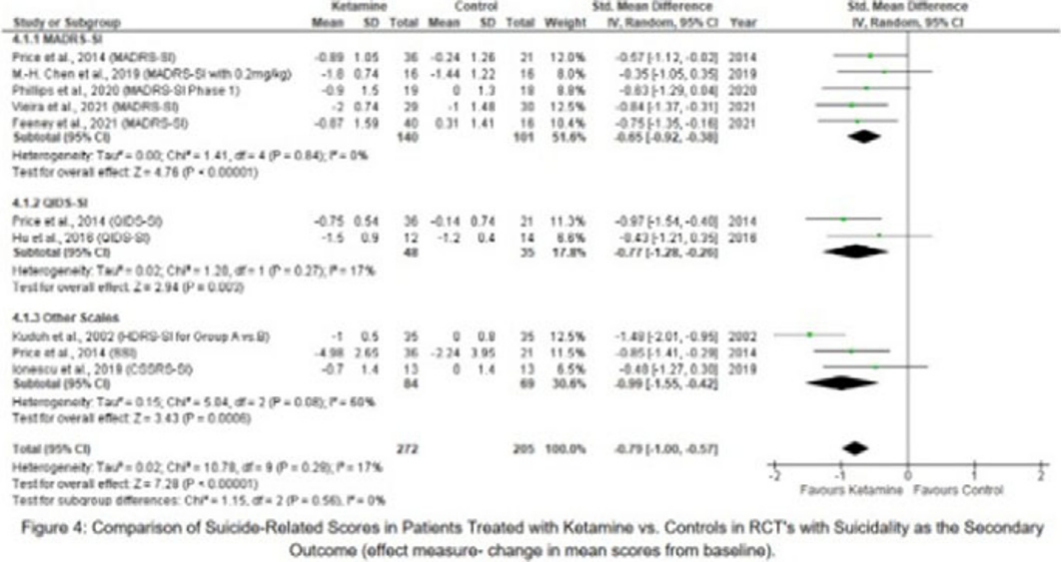
Image 2: Long description.

**Image 3:**
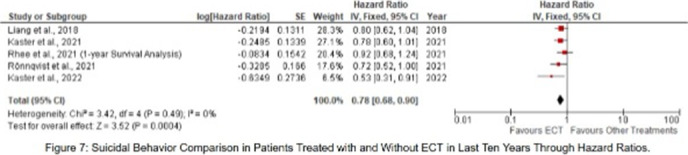
Image 3: Long description.

**Conclusions:**

Ketamine and ECT both have comparable efficacy in treating acute suicidality, despite different onset of action. The decision between ketamine and ECT should be individualized, guided by clinical urgency, patient preference, and contraindications.

**Disclosure of Interest:**

None Declared

## Long descriptions

### Image 1: Long description

The flowchart is divided into five vertical stages labeled on the left axis.

1. Identification and Retrieval. A top box shows PubMed searches using Me S H terms for Suicide and Ketamine (N equals 382) and Suicide and E C T (N equals 534), totaling N equals 916.

2. Duplicate Removal. From the total, 21 duplicates are removed, leaving 895 records.

3. Screening and Eligibility. 843 records are excluded, leaving 52 records included. A blue box on the right lists the Basis of Inclusion as original research and meta-analysis in humans in English. A second blue box lists the Basis of Exclusion: Title or Abstract (405), Non-original (359), Non-English (74), Post-Mortem (3), and Retracted Article (2), totaling 843.

4. Secondary Sources. 50 additional records are identified through references from primary sources and targeted Google Scholar searches for Ketamine, Esketamine, E C T, and suicide.

5. Inclusion. The final number of included records is 102 (52 primary plus 50 secondary). A table breaks these down into three subsections:

* Ketamine's Efficacy for Suicidality: 22 Primary, 08 Secondary, Total 30.

* E C T's Effect on Suicidality Subsection: 29 Primary, 28 Secondary, Total 57.

* Comparison of the Two Modalities Subsection: 01 Primary, 14 Secondary, Total 15.

The final total across all subsections is 52 Primary, 50 Secondary, and 102 overall.

Navigate back to Image 1:.

### Image 2: Long description

A forest plot divided into three subgroups: 4.1.1 M A D R S-S I, 4.1.2 Q I D S-S I, and 4.1.3 Other Scales.

Subgroup 4.1.1 M A D R S-S I includes five studies (Price et al. 2014, Chen et al. 2019, Phillips et al. 2020, Vieira et al. 2021, and Feeney et al. 2021). The subtotal Std. Mean Difference is -0.65 with a 95% C I of [-0.92, -0.38]. Heterogeneity: I-squared equals 0%.

Subgroup 4.1.2 Q I D S-S I includes two studies (Price et al. 2014 and Hu et al. 2016). The subtotal Std. Mean Difference is -0.77 with a 95% C I of [-1.28, -0.26]. Heterogeneity: I-squared equals 17%.

Subgroup 4.1.3 Other Scales includes three studies (Kudoh et al. 2002, Price et al. 2014, and Ionescu et al. 2019). The subtotal Std. Mean Difference is -0.99 with a 95% C I of [-1.55, -0.42]. Heterogeneity: I-squared equals 60%.

The overall total for all 10 studies shows a pooled Std. Mean Difference of -0.79 with a 95% C I of [-1.00, -0.57].

The visual plot on the right features a vertical line of no effect at 0. All individual study markers (green squares) and subgroup/total diamonds (black) are positioned to the left of the zero line, indicating a statistically significant effect favoring Ketamine. The x-axis ranges from -2 to 2, labeled Favours Ketamine on the left and Favours Control on the right.

Navigate back to Image 2:.

### Image 3: Long description

The forest plot contains a table with six columns: Study or Subgroup, log Hazard Ratio, S E, Weight, Hazard Ratio I V Fixed 95% C I, and Year.

Data rows from top to bottom:

* Liang et al., 2018: log Hazard Ratio -0.2194, S E 0.1311, Weight 28.3%, Hazard Ratio 0.80 [0.62, 1.04].

* Kaster et al., 2021: log Hazard Ratio -0.2485, S E 0.1339, Weight 27.1%, Hazard Ratio 0.78 [0.60, 1.01].

* Rhee et al., 2021 (1-year Survival Analysis): log Hazard Ratio -0.0834, S E 0.1642, Weight 20.4%, Hazard Ratio 0.92 [0.68, 1.24].

* Rönnqvist et al., 2021: log Hazard Ratio -0.3285, S E 0.186, Weight 17.6%, Hazard Ratio 0.72 [0.52, 1.00].

* Kaster et al., 2022: log Hazard Ratio -0.6349, S E 0.2736, Weight 6.5%, Hazard Ratio 0.53 [0.31, 0.91].

Total (95% C I): Weight 100.0%, Hazard Ratio 0.78 [0.68, 0.90].

Heterogeneity: Chi-squared = 3.42, d f = 4 (P = 0.49), I-squared = 0%.

Test for overall effect: Z = 3.52 (P = 0.0004).

The graphical section on the right shows a horizontal axis ranging from 0.01 to 100 on a log scale. A vertical line of no effect is at 1.0. Points to the left of 1.0 are labeled Favours E C T and points to the right are labeled Favours Other Treatments. Each study is represented by a red square with horizontal error bars. The pooled result is represented by a black diamond centered at 0.78, entirely to the left of the line of no effect.

Navigate back to Image 3:.

